# NFIB-Mediated lncRNA PVT1 Aggravates Laryngeal Squamous Cell Carcinoma Progression via the miR-1301-3p/MBNL1 Axis

**DOI:** 10.1155/2021/8675123

**Published:** 2021-11-12

**Authors:** Tian Tang, Feng Zeng

**Affiliations:** ^1^Department of Oncology, Renmin Hospital of Wuhan University, Wuhan, Hubei, China; ^2^Department of Otolaryngology-Head and Neck Surgery, Renmin Hospital of Wuhan University, Wuhan, Hubei, China

## Abstract

Laryngeal squamous cell carcinoma (LSCC) is one of the most common malignant tumors of head and neck cancers. In the past decades, although the therapy strategies of LSCC have made considerable improvement, the terrible outcomes of LSCC still bring an enormous burden to the world health care system. Novel therapeutic targets for LSCC are urgently needed. lncRNAs exert important roles in various biological progressions, including LSCC. Here, we aimed to investigate the function of lncRNA PVT1 in LSCC progression and its underlying molecular mechanisms. By conducting multiple experiments, our results showed that lncRNA PVT1 was upregulated in LSCC cell lines and regulated LSCC cell proliferation, apoptosis, and its cell susceptibility to natural killer (NK) cells. Moreover, it was found that lncRNA PVT1 promotes MBNL1 expression to regulate LSCC cellular progression through sponging miR-1301-3p. Our study might provide novel targets for LSCC basic research or clinical management.

## 1. Introduction

Laryngeal squamous cell carcinoma (LSCC), as one of the most common cancer types of head and neck malignancies [1], has become an enormous burden to the world health care system. Although significant improvements in LSCC clinical intervention, including surgery, chemotherapy, and radiotherapy, have been made in the past decades, the outcomes of LSCC patients remain poor [2]. Understanding the mechanisms underlying the development of LSCC is urgently needed for developing novel therapeutic targets.

Long noncoding RNAs (lncRNAs), as one subtype of noncoding RNAs (ncRNAs), possess more than 200 nucleotides. Its molecular functions have been in-depth studied recently. It has been revealed that lncRNAs are capable of transcriptionally and posttranscriptionally regulating gene expression [3, 4]. Moreover, lncRNAs were found to participate in various biological progressions, such as autophagy [5], immune response [6], inflammation diseases [7], and tumorigenesis [8], and modulate multiple cellular processes, including proliferation, migration, invasion, and apoptosis [9, 10].

Recently, accumulating evidence indicated that lncRNAs play an important role in the initiation or progression of LSCC [11–13]. Cao et al. elucidated that lncRNA IGKJ2-MALLP2 suppresses LSCC through interacting with miR-1911-3p to regulate p21 expression [14]. Xu et al. revealed that lncRNA HULC regulates LSCC development through modulating PTPRO [15]. Li et al. demonstrated the function of lncRNA SNHG20/miR-140 in the progression of LSCC [16]. All those research suggested that lncRNAs have a promising prospect to be novel LSCC therapeutic targets.

In the current study, we aimed to investigate the biological function of lncRNA PVT1 in LSCC development. A previous study has reported that lncRNA PVT1 was upregulated in LSCC tissues and promoted LSCC progression. However, its underlying mechanisms demand further study [17]. Here, we found that the expression level of lncRNA PVT1 in LSCC cell lines was upregulated. Next, by conducting a serial *in vitro* experiment, our results showed that lncRNA PVT1 regulates LSCC proliferation, apoptosis, and NK cell-mediated cytotoxicity towards LSCC cells. Through investigating the underlying molecular mechanism, a novel lncRNA PVT1/miR-1301-3p/MBNL1 axis was revealed. Furthermore, we elucidated that lncRNA PVT1 expression in LSCC cells could be regulated by NFIB. Our research might provide a new insight for LSCC therapeutic target research.

## 2. Methods and Materials

### 2.1. Cell Culture and Transfection

Cells used in the current study, including HEK-293T, HOK, MRC-5, FD-LSC-1, and TU-177, were commercially obtained from the Cell Bank of the Chinese Academy of Sciences. FD-LSC-1 cells were cultured in the Bronchial Epithelial Cell Growth Medium (Lonza, USA) added with 10% FBS and supplemented with penicillin/streptomycin in a 5% CO_2_ environment at 37°C. HEK-293T, HOK, MRC-5, and TU-177 cells were cultured using DMEM added with 10% FBS and supplemented with penicillin/streptomycin in a 5% CO_2_ environment at 37°C. All cell lines were tested using the TransDetect PCR Mycoplasma Detection Kit (TransGen Biotech, China). All siRNAs, lentiviruses, and plasmids were synthesized and purchased from GenePharma, Shanghai, China. The Lipofectamine 3000 Reagent (Thermo Fisher Scientific, USA) was used to perform all transfections following the manufacturer's protocols. The information of miR-1301-3p is as follows: miR-1301-3p mimic: 5′-UUG CAG CUG CCU GGG AGU GAC UUC-3′; miR-1301-3p inhibitor: 5′-GAA GUC ACU CCC AGG CAG CUG CAA-3′; and miR-1301-3p negative control (NC): 5′-UUC UCC GAA CGU GUC ACG UTT-3′.

### 2.2. qRT-PCR

Total RNAs from LSCC tissue samples and cell lines were isolated using the TRIzol reagent (Invitrogen, USA) according to the manufacturer's protocol. The SuperScript IV First-Strand Synthesis System (Invitrogen, CA) was used to conduct the reverse transcription process of miRNA and lncRNA following the manufacturer's instruction. With GAPDH as the internal control, the qualification of RNAs was conducted using the 2^-△△Ct^ method. All primers used in the current study are as follows (Table 1).

### 2.3. Western Blotting

Proteins were isolated from cells using the RIPA protein extraction reagent (Beyotime, China) and added with a protease inhibitor cocktail (Roche, USA) in accordance with protocols. Collected proteins were separated by SDS-PAGE gels and electrically transferred onto polyvinylidene fluoride (PVDF) membranes (Millipore, USA). Subsequently, membranes were incubated with primary antibodies overnight at 4°C. Next, membranes were washed by TBST three times and then subjected to second antibodies for 2 h at room temperature. A Fusion FX5 image analysis system (Vilber Lourmat, France) was used to visualize the results. Antibodies used in the current study are as follows: MBNL1 (sc-47740, 1 : 1000, Santa Cruz Biotechnology) and actin (ab8226, 1 *μ*g/mL, Abcam).

### 2.4. CCK-8

The proliferation levels of pretreated LSCC cells were measured using the Cell Counting Kit 8 (CCK-8) (TransGen Biotech, China) according to the manufacturer's protocol. Briefly, infected cells were digested and seeded in a 96-well plate (1∗10^4^/well). After that, cells were added with 10 *μ*L CCK-8 dilutions at 0 h, 24 h, 48 h, 72 h, and 96 h for half an hour. Then, treated cells were subjected to a SpectraMax i3x multifunctional microplate detection system (Molecular Devices, CA) for absorbance detection at 450 nm. Experiments were repeated three times, and results were recorded.

### 2.5. Cell Colony Formation Assay

Collectively, cells were seeded in a 35 mm dish at a density of 1000 cells per well after transfection. Next, cells were cultured in the dishes for 2 weeks. After that, cells were washed by PBS and added with 4% paraformaldehyde for 30 min. Next, 0.1% crystal violet solution was applied to stain cells at room temperature for 15 min. Results were visualized by the image capturing.

### 2.6. Apoptosis Analysis

Cell apoptosis analysis was conducted using an Annexin V-fluorescein isothiocyanate (FITC)/propidium iodide (PI) cell apoptosis kit (Invitrogen) following the manufacturer's instruction. Briefly, pretransfected cells were collected from each group and washed by PBS three times. Next, cells were supplied with 5 *μ*L Annexin V/FITC and 5 *μ*L PI for 10 min at 25°C. Then, results were analyzed using FACScan flow cytometry (BD Biosciences, USA). Experiments were conducted three times, and results were recorded. The apoptotic rate of indicated cells was calculated using the sum value of the right upper results plus the right lower results.

### 2.7. lncRNA Pull-Down Assay

An *in vitro* transcription kit from Thermo Fisher Scientific (K0441) was used to acquire the full-length sense or antisense of PVT1 following the manufacturer's instruction. Subsequently, RNA Pull-Down assay was performed using a Magnetic RNA-Protein Pull-Down Kit (Thermo Fisher Scientific) according to the protocol. Biotinylated probes were synthesized and obtained from GeneChem (Shanghai, China) Company. Experiments were conducted according to published research [18]. Experiments were conducted in triplicate.

### 2.8. Chromatin Immunoprecipitation (ChIP)

A ChIP kit (CST, USA) was applied to conduct ChIP assays in the current study according to the instructions provided by the manufacturer. In summary, formaldehyde was subjected to cells for crosslinking. Subsequently, cells were sonicated to a length between 200 and 1000 bp. Next, the anti-IgG (CST, #3900S, 2.5 mg/mL) antibody and anti-NFIB (Abcam, ab186738, 1 : 30) antibody were used to perform immunoprecipitation. Results were analyzed by the qRT-PCR assay.

### 2.9. Dual-Luciferase Reporter Assay

The interaction between miR-1301-3p and lncRNA PVT1 or MBNL1 was validated by transfecting empty pmirGLO-NC, pmirGLO-PVT1-WT (pmirGLO-MBNL1-WT), or pmirGLO-PVT1-MUT (pmirGLO-MBNL1-MUT) with miR-1301-3p mimics or NC mimics into HEK-293T, FD-LSC-1, or TU-177 cells as indicated. Transfections were accomplished using a Lipofectamine 2000 (Invitrogen) solution. A Dual-Luciferase Reporter Assay System (Promega) was used to measure experiment results. Relative luciferase activity was normalized to Renilla luciferase activity. The experiment was performed in triplicate.

### 2.10. Calcein Release Experiment

Firstly, NK cells were incubated with cells prestained with 35 *μ*M calcein-AM (Dojindo) at indicated *E*/*T*ratios. Subsequently, cells were collected and seeded in a 96-well plate for 3 h, and the supernatant was collected for fluorescent detection. The result of the fluorescent examination was considered a spontaneous release value. Next, the maximum release value was recorded by calculating the fluorescent examination results of the supernatant of the cells pretreated with a 2% Triton X-100-containing medium. Results were analyzed using (test release value − spontaneous release value)/(maximum release value − spontaneous release value).

### 2.11. Perforin Polarization Experiment

NK cells were subjected to treated LSCC cells for coculturing. Half an hour later, cocultured cells were collected and seeded in a 12-well plate and added with poly-D-lysine-coated slides for 1 h. After that, slides were stained with 4% paraformaldehyde and antibodies. Results were recorded using a confocal microscope.

### 2.12. Conjugation Experiment

Firstly, NK cells were incubated with cells prestained with 35 *μ*M calcein-AM (Dojindo) at indicated *E*/*T*ratios (2 : 1). Cocultured cells were harvested at the indicated time for the flow cytometry experiment. Results were calculated by [the double − positive events]/[the total events]. Experiments were repeated three times.

### 2.13. Statistical Analysis

All statistical analyses in the current study were calculated using SPSS (23.0V, USA) or Prism 9 software (7.0V, USA). All experiments were performed at least three times. Statistical differences between the two groups were analyzed using Student's *t*-test. The comparison among three or more groups was analyzed using the one-way ANOVA method. All results were presented as the mean ± standard deviation (SD). ^∗^*P* < 0.05 was considered statistically significant.

## 3. Results

### 3.1. The Functional Role of lncRNA PVT1 in LSCC Progression

**Figure 1:**
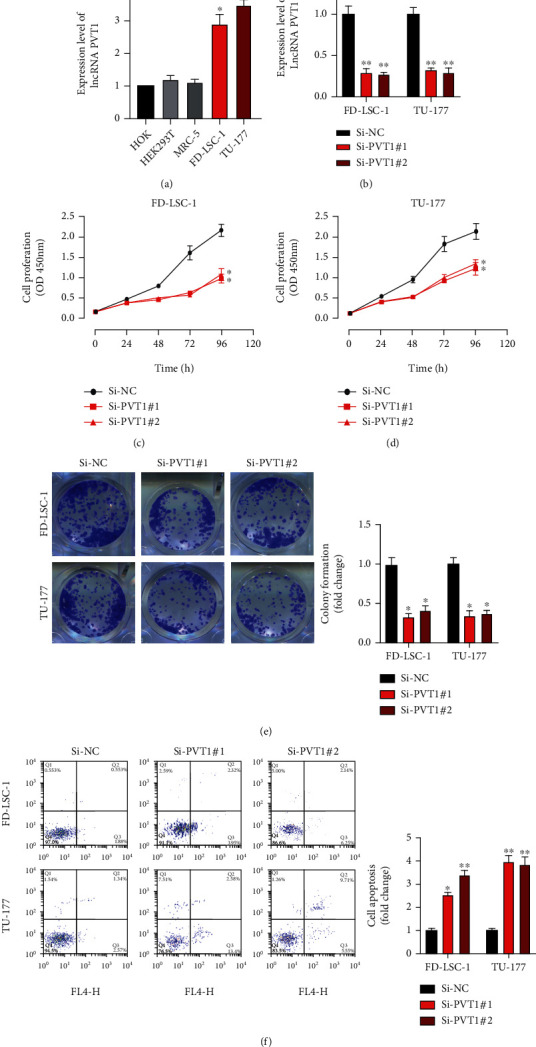
The functional role of lncRNA PVT1 in LSCC progression. (a) The expression of lncRNA PVT1 in LSCC tumor tissues. (b) The expression of lncRNA PVT1 in LSCC cells. (c) Cells were transfected with Si-NC, Si-PVT1#1, and Si-PVT1#2. (d, e) Cell proliferation levels were detected using CCK-8 and cell colony assays. (f) Cell apoptotic rates were detected using flow cytometry. All experiments were repeated three times. ^∗^*P* < 0.05, ^∗∗^*P* < 0.01.

To investigate whether lncRNA PVT1 participates in LSCC progression, firstly, we examined the expression level of PVT1 in LSCC cell lines. Results of qRT-PCR showed that PVT1 expression in FD-LSC-1 and TU-177 was significantly upregulated compared with that in normal cell line HOK (Figure 1(a)). Thereafter, PVT1 knockdown cell models were generated as indicated to elucidate the biological function of PVT1 in LSCC cellular progression (Figure 1(b)). Next, results of CCK-8 (Figure 1(c)) and colony formation assays (Figure 1(d)) showed that downregulated PVT1 suppressed LSCC cell proliferation levels. Cell apoptosis detection results showed that PVT1 knockdown increased LSCC cell apoptotic rates (Figure 1(e)). Based on those results, we conclude that lncRNA PVT1 participates in LSCC progression.

### 3.2. lncRNA PVT1 Regulates the Natural Killer Cell-Mediated Cytotoxicity to LSCC Cells

**Figure 2:**
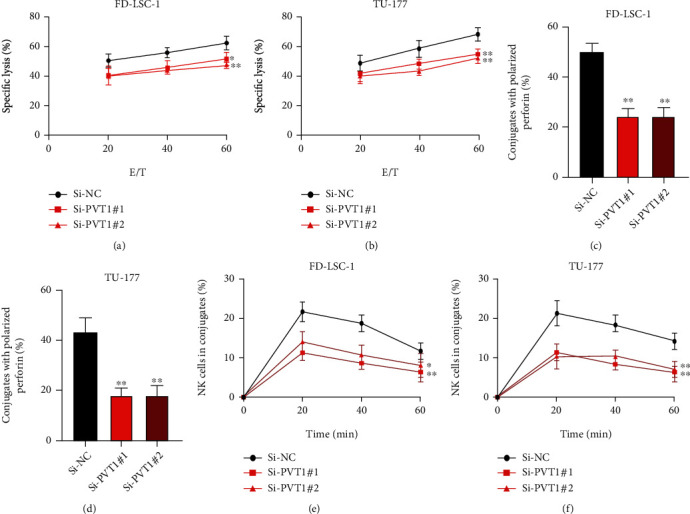
lncRNA PVT1 regulates the natural killer cell-mediated cytotoxicity to LSCC cells. (a, b) NK cell cytotoxicity to LSCC cells was evaluated using calcein release assay. (c, d) Perforin polarization assay was conducted to measure the perforin-containing NK cells, which were against lncRNA PVT1 knockdown LSCC cells. (e, f) Conjugation assay was applied to measure the conjugate formation between NK cells and lncRNA PVT1 knockdown LSCC cells. All assays were conducted three times. ^∗^*P* < 0.05, ^∗∗^*P* < 0.01.

Previous studies have demonstrated that both the linear and circular forms of PVT1 play an important role in the progression of the immune response [19]. However, little is known about the function of lncRNA PVT1 in the immune system. It has been well documented that the NK cell, as an innate antitumor immune response character, exerts essential roles in various tumorigeneses [20–22]. To determine whether lncRNA PVT1 plays its role in LSCC progression via influencing the susceptibility of NK cells, calcein release assay (Figures 2(a) and 2(b)), perforin polarization assay (Figures 2(c) and 2(d)), and conjugation assay (Figures 2(e) and 2(f)) using lncRNA PVT1 knockdown LSCC cells were conducted. Interestingly, we found that lncRNA knockdown repressed the NK cell-mediated cytotoxicity towards LSCC cells. From those results, we conclude that lncRNA PVT1 plays its biological function in the interaction of LSCC cells and NK cells.

### 3.3. lncRNA PVT1 Sponges miR-1301-3p

**Figure 3:**
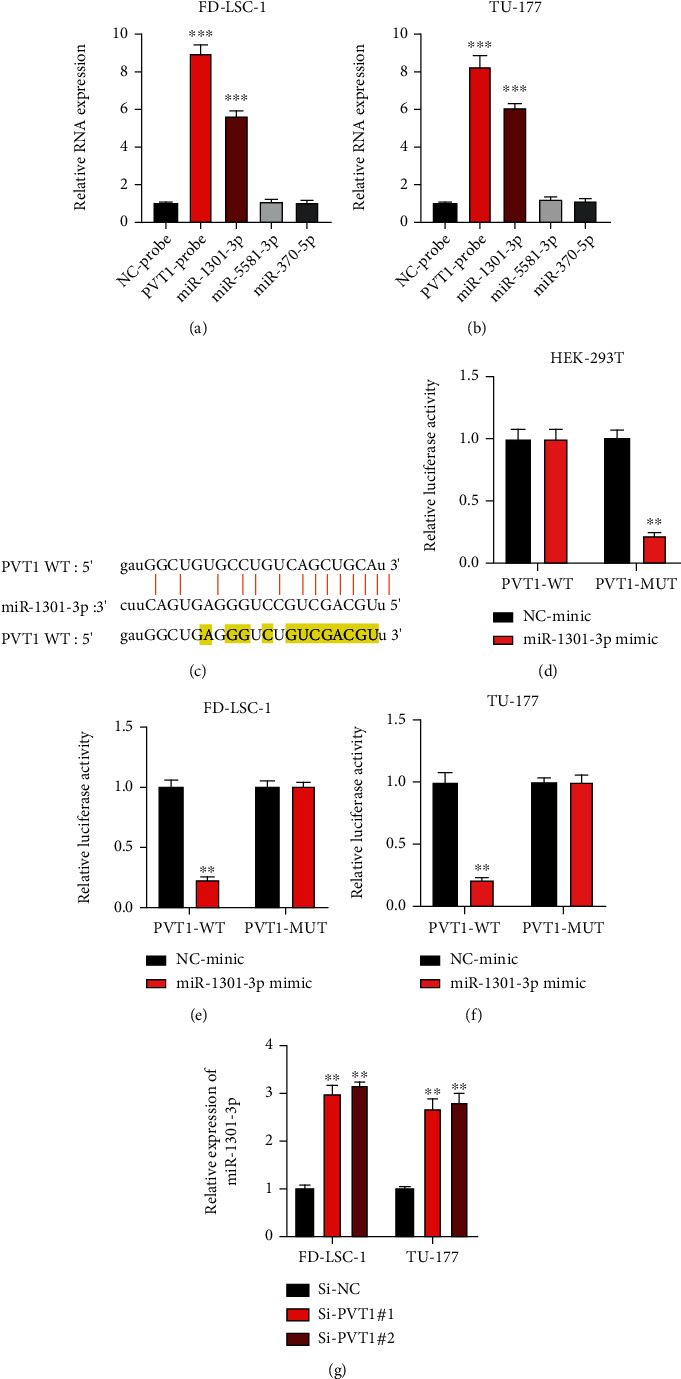
lncRNA PVT1 sponges miR-1301-3p. (a, b) FD-LSC-1 (a) and TU-177 (b) cells were infected with bio-NC and bio-PVT1 probes. Relative expressions of putative miRNA targets were measured by qRT-PCR. (c) Prediction of the binding sites between lncRNA PVT1 and miR-1301-3p was shown. (d–f) Dual-luciferase assay was performed to measure the association between lncRNA PVT1 and miR-1301-3p in HEK-293T (d), FD-LSC-1 (e), and TU-177 (f) cells. (g) The expression of miR-1301-3p in LSCC cells upon lncRNA PVT1 downregulation was measured by qRT-PCR. The above experiments were performed three times. ^∗∗^*P* < 0.01, ^∗∗∗^*P* < 0.001.

The above results showed that lncRNA PVT1 modulates LSCC progression by regulating LSCC cellular progression and cell susceptibility to NK cells. Subsequently, its underlying mechanisms were investigated. By utilizing bioinformatic analysis (ENCORI: http://starbase.sysu.edu.cn/, with CLIP Data strict stringency (≥5)), three putative miRNA targets of lncRNA PVT1 were found. Biotinylated RNA probes were constructed and stably transfected into FD-LSC-1 and TU-177 cells. As shown in Figures 3(a) and 3(b), miR-1301-3p was significantly enriched. Bioinformatic prediction of the binding sites between lncRNA PVT1 and miR-1301-3p was presented (Figure 3(c)). Next, a dual-luciferase reporter gene assay was performed to assess the interaction between lncRNA PVT1 and miR-1301-3p. As shown in Figures 3(d)–3(f), lncRNA PVT1 directly targets miR-1301-3p. Furthermore, the expression of miR-1301-3p was negatively regulated by lncRNA PVT1 in LSCC cells (Figure 3(g)). Those results suggest that lncRNA PVT1 acts as a molecular sponge of miR-1301-3p in LSCC cells.

### 3.4. miR-1301-3p Regulates LSCC Proliferation and Impacts the Susceptibility of LSCC Cells to NK Cells

**Figure 4:**
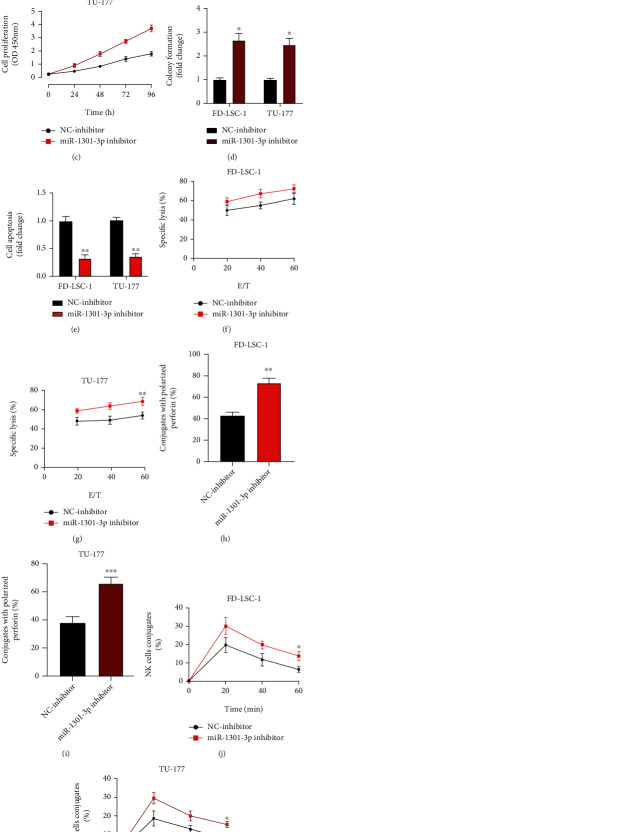
miR-1301-3p regulates LSCC proliferation and impacts the susceptibility of LSCC cells to NK cells. (a) FD-LSC-1 and TU-177 cells were stably infected with the NC inhibitor and miR-1301-3p inhibitor to generate miR-1301-3p knockdown cell models; transfection efficiency was measured by qRT-PCR. (b–d) Cell proliferation levels were detected using CCK-8 (b, c) and cell colony assays (d). (e) Cell apoptotic rates were detected using flow cytometry. (f, g) NK cell cytotoxicity to miR-1301-3p knockdown LSCC cells was measured by calcein release assay. (h, i) Perforin polarization assay was conducted to measure the perforin-containing NK cells, which were against miR-1301-3p knockdown LSCC cells. (j, k) Conjugation assay was performed to detect the conjugate formation between NK cells and miR-1301-3p knockdown LSCC cells. All assays were conducted three times. ^∗^*P* < 0.05, ^∗∗^*P* < 0.01.

We have demonstrated that miR-1301-3p is a downstream target of lncRNA PVT1 in LSCC cells. However, the biological functions of miR-1301-3p in LSCC cells have not been studied. Therefore, we conducted loss-of-function experiments using miR-1301-3p downregulated LSCC cells (Figure 4(a)). Results of CCK-8 (Figures 4(b) and 4(c)), cell colony formation (Figure 4(d)), and flow cytometry (Figure 4(e)) assays showed that miR-1301-3p knockdown markedly promoted cell proliferation and repressed cell apoptotic rates. Moreover, miR-1301-3p knockdown significantly decreased the LSCC cell susceptibility to NK cells (Figures 4(f)–4(k)). The gain-of-function experiments using miR-1301-3p overexpression LSCC cells were also performed; miR-1301-3p overexpression significantly inhibited cell proliferation and promoted cell apoptotic rates. Further, miR-1301-3p overexpression markedly increased the LSCC cell susceptibility to NK cells (Fig. S). Based on those results, we conclude that miR-1301-3p plays a tumor suppressor role in LSCC progression. Interestingly, miR-1301-3p overexpression attenuates the NK cell-mediated cytotoxicity towards LSCC cells.

### 3.5. MBNL1 Is a Downstream Target of miR-1301-3p

**Figure 5:**
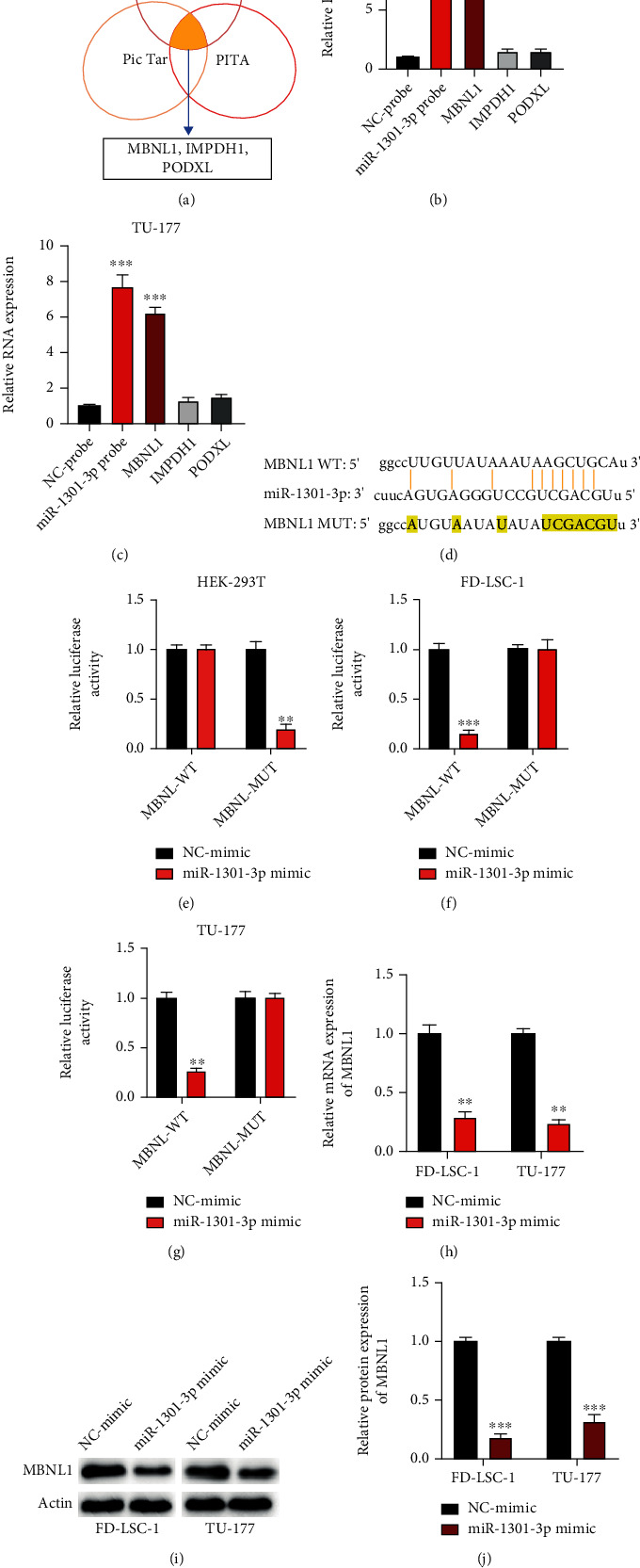
MBNL1 is a downstream target of miR-1301-3p. (a) Prediction of miR-1301-3p targets of bioinformatic analysis. (b, c) RIP assay was utilized to examine the interaction between MBNL1 and miR-1301-3p. (d) The wild-type and mutant-type binding sites between miR-1301-3p and MBNL1 were shown. (e–g) Dual-luciferase assay was performed to measure the association between MBNL1 and miR-1301-3p in HEK-293T (e), FD-LSC-1 (f), and TU-177 (g) cells. (h–j) MBNL1 expression upon miR-1301-3p overexpression was detected by qRT-PCR (h) and western blotting (i, j). The above experiments were performed at least three times. ^∗∗^*P* < 0.01, ^∗∗∗^*P* < 0.001.

Next, by utilizing miRmap (https://mirmap.ezlab.org/), PicTar (https://pictar.mdc-berlin.de/), and PITA (https://genie.weizmann.ac.il/pubs/mir07/mir07_data.html) datasets, three putative downstream factors of miR-1301-3p were identified (Figure 5(a)). RIP assay using biotinylated miR-1301-3p probes was applied. It was found that MBNL1 was abundantly enriched in miR-1301-3p probes compared with the normal control in both the FD-LSC-1 and TU-177 cells (Figures 5(b) and 5(c)). The predicted binding sites between miR-1301-3p and MBNL1 are shown in Figure 5(d). The interaction was confirmed by dual-luciferase reporter gene assay (Figures 5(e)–5(g)). Furthermore, the expression of MBNL1 in LSCC cells was negatively regulated by miR-1301-3p (Figures 5(h)–5(j)).

### 3.6. lncRNA PVT1 Regulates LSCC Progression via the miR-1301-3p/MBNL1 Axis

**Figure 6:**
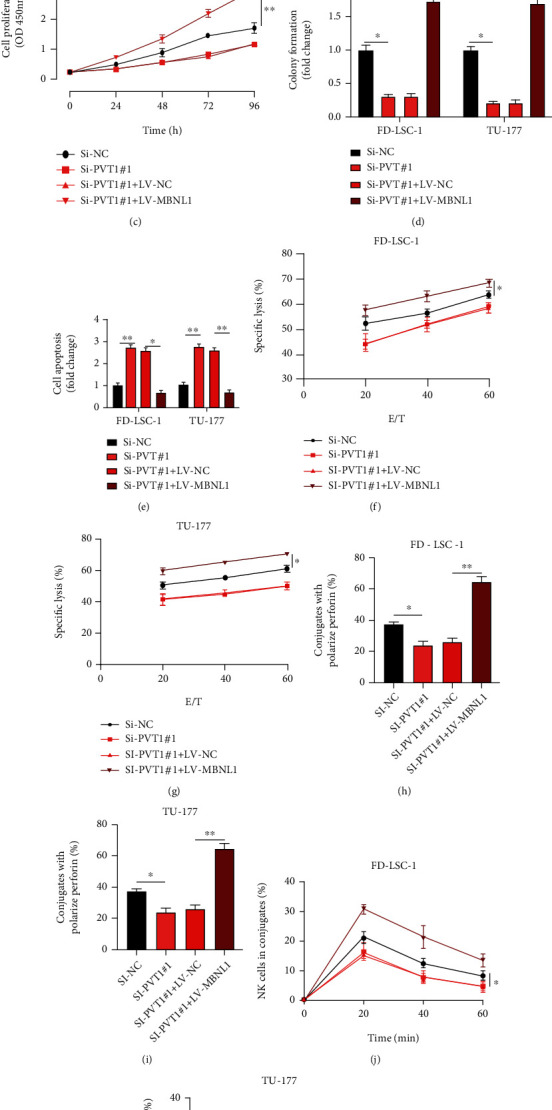
lncRNA PVT1 regulates LSCC progression via the miR-1301-3p/MBNL1 axis. (a) FD-LSC-1 and TU-177 cells were infected with Si-NC, Si-PVT1#1, Si-PVT1#1**+**LV-NC, and Si-PVT1#1**+**LV-MBNL1; relative expressions of MBNL1 were detected. (b–d) Cell proliferation levels were detected using CCK-8 (b, c) and cell colony assays (d). (e) Cell apoptotic rates were detected using flow cytometry. (f, g) NK cell cytotoxicity to indicated LSCC cells was measured by calcein release assay. (h, i) Perforin polarization assay was conducted to measure the perforin-containing NK cells, which were against indicated LSCC cells. (j, k) Conjugation assay was performed to detect the conjugate formation between NK cells and indicated LSCC cells. All assays were conducted three times. ^∗^*P* < 0.05, ^∗∗^*P* < 0.01, and ^∗∗∗^*P* < 0.001.

Our study has demonstrated the molecular relationship between miR-1301-3p and lncRNA PVT1 or MBNL1. Thereafter, the biological role of the lncRNA PVT1/miR-1301-3p/MBNL1 axis in the LSCC cellular progression was investigated. Cell models were generated as indicated in Figure 6(a). The expression of MBNL1 was measured. Results of CCK-8 (Figures 6(b) and 6(c)), cell colony formation (Figure 6(d)), and cell apoptosis assays (Figure 6(e)) showed that the effect of lncRNA PVT1 knockdown on LSCC cellular progression was reversed by MBNL1 overexpression. Furthermore, MBNL1 overexpression rescued the inhibitive effect of lncRNA PVT1 knockdown on the susceptibility of LSCC cells to NK cells (Figures 6(f)–6(k)). Our study has partially elucidated the novel lncRNA PVT1/miR-1301-3p/MBNL1 axis in the progression of LSCC.

### 3.7. lncRNA Expression in LSCC Cells Is Induced by NFIB

**Figure 7:**
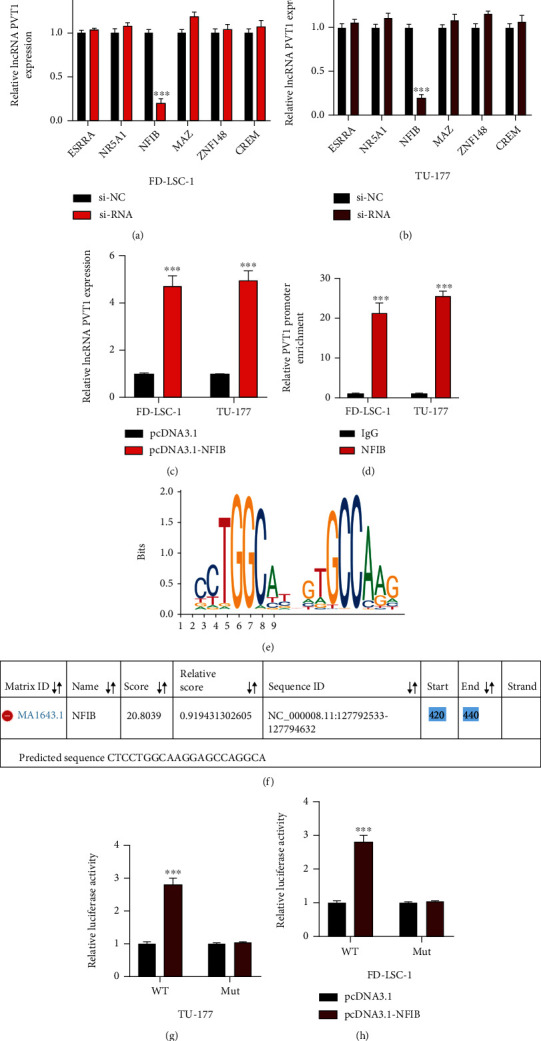
lncRNA expression in LSCC cells is induced by NFIB. (a, b) Relative expression level of lncRNA PVT1 in knockdown LSCC cell models pretransfected with indicated siRNAs was measured by qRT-PCR. (c) Relative lncRNA PVT1 expression in NFIB overexpression LSCC cells was detected by qRT-PCR. (d) ChIP assay using anti-IgG and anti-NFIB was performed to evaluate the molecular relationship between the PVT1 promoter and NFIB in LSCC cells; results were analyzed using the qRT-PCR assay. (e) The digraph binding sequence of NFIB was obtained from the JASPAR dataset. (f) The predicted binding region of the PVT1 promoter was obtained from the JASPAR dataset. (g, h) The molecular relationship between the PVT1 promoter and NFIB was assessed by luciferase reporter gene assay. All assays were conducted three times. ^∗∗∗^*P* < 0.001.

The phenomenon that lncRNA expression could be mediated by transcription factors has been reported in many studies [23, 24]. Huang et al. have elucidated that lncRNA PVT1 expression in lung cancer cells could be induced by transcription factor YY1. Here, we aimed to elucidate the upstream factor of lncRNA PVT1 in LSCC cells. Firstly, we used the JASPAR dataset (http://jaspar.genereg.net/) to predict putative transcription factors of lncRNA PVT1. Results predicted six potential factors of lncRNA PVT1 (ESRRA, NR5A1, NFIB, MAZ, ZNF148, and CREM). Through generating knockdown cell models for each factor, it was found that lncRNA PVT1 expression was positively regulated in Nuclear Factor I B (NFIB) up- or downregulated LSCC cells (Figures 7(a)–7(c)). Moreover, ChIP results showed that the PVT1 promoter was markedly enriched in anti-NFIB LSCC cells compared with anti-IgG (Figure 7(d)). The above results indicated that NFIB might be the transcription factor of lncRNA PVT1 in LSCC cells. Next, we obtained the putative binding sequences between NFIB (Figure 7(e)) and the PVT1 promoter (Figure 7(f)) from the JASPAR dataset. Results of dual-luciferase reporter gene assay showed that NFIB overexpression markedly increased the fluorescence intensity when cotransfected with PVT1-WT, not PVT1-MUT, suggesting that NFIB was directly binding with the PVT1 promoter in LSCC cells (Figures 7(g) and 7(h)). From the above results, we conclude that lncRNA PVT1 expression in LSCC cells could be transcriptionally mediated by NFIB.

## 4. Discussion

LSCC ranks as the second malignancy tumor of head and neck cancers. Due to its tendency of local invasion, metastasis, and chemotherapy resistance, its morbidity and mortality are constantly growing [25, 26]. It has been reported that the incidence of LSCC in China is near four times higher than that in the USA. However, with the development of clinical treatment strategies in recent years, the LSCC patients' survival rate is still low [27–29]. With the high-throughput technology innovation in the past two decades, the dysregulation and dysfunction of RNAs, especially lncRNAs, have got wildly scientific interest and are considered to be one auspicious direction for disease management. However, the underlying mechanisms of lncRNAs in LSCC development are still not fully understood.

In the current study, we aimed to demonstrate the function of lncRNA PVT1 and the molecular mechanisms underlying LSCC development. lncRNA PVT1 has been studied in multiple cancer types, including gastric cancer [30, 31], ovarian cancer [32], and pancreatic cancer [18]. And the molecular functions of lncRNA PVT1 in those cancers have been intensely investigated, which emphasized the important role of lncRNA PVT1 in various tumorigeneses. Published research from Zheng et al. has revealed that lncRNA PVT1 is upregulated in LSCC tumor tissues and promoted LSCC cellular progression via sponging miR-519d-3p [17]. However, our group decided to further explore the biological and mechanical role of lncRNA PVT1 in LSCC progression. Firstly, we confirmed that the expression level of lncRNA PVT1 in LSCC cell lines was upregulated. Next, we found that downregulated lncRNA PVT1 inhibited LSCC cell proliferation and promoted LSCC cell apoptotic rates. Moreover, dysregulation of lncRNA PVT1 is capable of influencing LSCC cell susceptibility to NK cells.

The NK cell is one of the most important members of the innate lymphoid cell family [33, 34]. Its essential role in cancer progression has been elucidated by multiple research [21, 35, 36]. Interestingly, our results showed that lncRNA PVT1 downregulation in LSCC cells increased its susceptibility to NK cells, making LSCC cells less venerable to NK cell-mediated cytotoxicity. However, its molecular mechanisms demand further study.

Our results have elucidated the downstream mechanisms of lncRNA PVT1 in LSCC progression. We also noticed that lncRNA PVT1 expression in lung cancer cells could be regulated by a transcription factor YY1 [37]. The important role of the transcription factor in lncRNA regulation is emerging [23, 24, 38]. To better understand the function of lncRNA PVT1, we investigated the upstream factor of lncRNA PVT1. Our results found that NFIB could regulate lncRNA PVT1 expression in LSCC cells through directly binding to the promoter region of PVT1. Our results renewed the molecular pattern of lncRNA PVT1 in LSCC.

Collectively, our study has partially demonstrated that lncRNA PVT1 regulates LSCC cell viability and impacts the susceptibility of LSCC cells to NK cells. Furthermore, a novel NFIB/lncPVT1/miR-1301-3p/MBNL1 axis has been partially elucidated in LSCC progression. Our study might provide a new insight for LSCC basic research and novel therapeutic targets for LSCC clinical intervention.

## Figures and Tables

**Table 1:** 

Gene		Sequence
PVT1	F	5′-CATCCGGCGCTCAGCT-3′
	R	5′-TCATGATGGCTGTATGTGCCA-3′

miR-1301-3p	F	5′-GCCGAGTTGCAGCTGCCTGGGA-3′
	R	5′-CTCAACTGGTGTCGTGGA-3′

miR-5581-3p	F	5′-CGTCTTGCAGGCCGTCATG-3′
	R	5′-GCTGTCAACGATACGCTACCTA-3′

miR-370-5p	F	5′-ACACTCCAGCTGGGCAGGTCACGTCTCTGC-3′
	R	5′-CTCAACTGGTGTCGTGGAGTCGGCAATTCAGTTGAGGTAACTGC-3′

PODXL	F	5′-AACCCGGCCCAAGATAAGTG-3′
	R	5′-TTGGCAGGGAGCTTAGTGTG-3′

IMPDH1	F	5′-CGACACCCCGTTCAGAACTAT-3′
	R	5′-GCTGATCAGGTAGTCCGCC-3′

MBNL1	F	5′-CTTGCTCACGACCAGAC-3′
	R	5′-ATTCCGCCCATTTATC-3′

NFIB	F	5′-CTTATCCAATCCCGACCAGA-3′
	R	5′-GACTAGATCCAGACGCCAGACT-3′

## Data Availability

The data performed to support the findings of this study are included within the article.

## Supplementary Materials

Supplementary Materials Figure S: miR-1301-3p regulates LSCC proliferation and impacts the susceptibility of LSCC cells to NK cells. (A) FD-LSC-1 and TU-177 cells were stably infected with NC mimics and miR-1301-3p mimics to generate miR-1301-3p overexpression cell models; transfection efficiency was measured by qRT-PCR. (B–D) Cell proliferation levels were detected using CCK-8 (B, C) and cell colony assays (D). (E) Cell apoptotic rates were detected using flow cytometry. (F, G) NK cell cytotoxicity to miR-1301-3p overexpression LSCC cells was measured by calcein release assay. (H, I) Perforin polarization assay was conducted to measure the perforin-containing NK cells, which were against miR-1301-3p overexpression LSCC cells. (J, K) Conjugation assay was performed to detect the conjugate formation between NK cells and miR-1301-3p overexpression LSCC cells. All assays were conducted three times. ^∗^*P* < 0.05, ^∗∗^*P* < 0.01.
