# Supplementary material for: NFIB-Mediated lncRNA PVT1 Aggravates Laryngeal Squamous Cell Carcinoma Progression via the miR-1301-3p/MBNL1 Axis
Source: J Immunol Res. 2021 Nov 12;2021:8675123. doi: 10.1155/2021/8675123 (PMC8604577; doi:10.1155/2021/8675123)
Supplement: Supplementary Materials — Figure S: miR-1301-3p regulates LSCC proliferation and impacts the susceptibility of LSCC cells to NK cells. (A) FD-LSC-1 and TU-177 cells were stably infected with NC mimics and miR-1301-3p mimics to generate miR-1301-3p overexpression cell models; transfection efficiency was measured by qRT-PCR. (B–D) Cell proliferation levels were detected using CCK-8 (B, C) and cell colony assays (D). (E) Cell apoptotic rates were detected using flow cytometry. (F, G) NK cell cytotoxicity to miR-1301-3p overexpression LSCC cells was measured by calcein release assay. (H, I) Perforin polarization assay was conducted to measure the perforin-containing NK cells, which were against miR-1301-3p overexpression LSCC cells. (J, K) Conjugation assay was performed to detect the conjugate formation between NK cells and miR-1301-3p overexpression LSCC cells. All assays were conducted three times. ∗P < 0.05, ∗∗P < 0.01. [file 8675123.f1.docx]

Supplementary figure S.


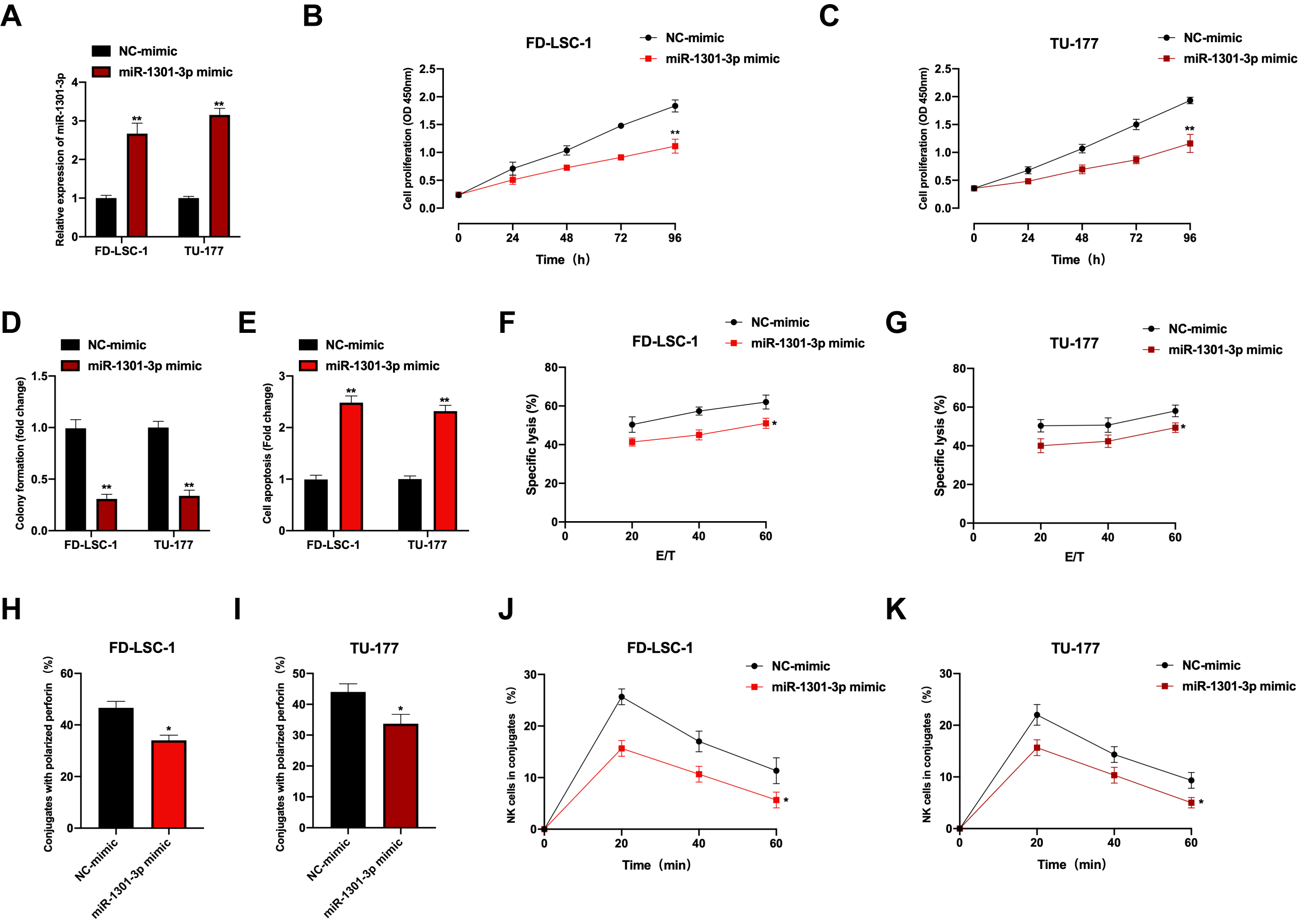


Figure S. MiR-1301-3p regulates LSCC proliferation and impacts the susceptibility of LSCC cells to NK cells. A: FD-LSC-1 and TU-177 cells were stably infected with NC-mimic and miR-1301-3p mimic to generate miR-1301-3p overexpression cell models, transfection efficiency was measured by qRT-PCR. B-D: Cell proliferation levels were detected using CCK-8 (B and C), and cell colony assays (D). E: Cell apoptotic rates were detected using flow cytometry. F and G: NK cell cytotoxicity to miR-1301-3p overexpression LSCC cells was measured by calcein release assay. H and I: perforin polarization assay was conducted to measure the perforin-containing NK cells, which against miR-1301-3p overexpression LSCC cells. J and K: Conjugation assay was performed to detect the conjugate formation between NK cells and miR-1301-3p overexpression LSCC cells. All assays were conducted three times. **P* < 0.05, ***P* < 0.01.
